# Core Genome Multilocus Sequence Typing for Identification of Globally Distributed Clonal Groups and Differentiation of Outbreak Strains of Listeria monocytogenes

**DOI:** 10.1128/AEM.01532-16

**Published:** 2016-09-30

**Authors:** Yi Chen, Narjol Gonzalez-Escalona, Thomas S. Hammack, Marc W. Allard, Errol A. Strain, Eric W. Brown

**Affiliations:** Center for Food Safety and Applied Nutrition, U.S. Food and Drug Administration, College Park, Maryland, USA; Pennsylvania State University

## Abstract

Many listeriosis outbreaks are caused by a few globally distributed clonal groups, designated clonal complexes or epidemic clones, of Listeria monocytogenes, several of which have been defined by classic multilocus sequence typing (MLST) schemes targeting 6 to 8 housekeeping or virulence genes. We have developed and evaluated core genome MLST (cgMLST) schemes and applied them to isolates from multiple clonal groups, including those associated with 39 listeriosis outbreaks. The cgMLST clusters were congruent with MLST-defined clonal groups, which had various degrees of diversity at the whole-genome level. Notably, cgMLST could distinguish among outbreak strains and epidemiologically unrelated strains of the same clonal group, which could not be achieved using classic MLST schemes. The precise selection of cgMLST gene targets may not be critical for the general identification of clonal groups and outbreak strains. cgMLST analyses further identified outbreak strains, including those associated with recent outbreaks linked to contaminated French-style cheese, Hispanic-style cheese, stone fruit, caramel apple, ice cream, and packaged leafy green salad, as belonging to major clonal groups. We further developed lineage-specific cgMLST schemes, which can include accessory genes when core genomes do not possess sufficient diversity, and this provided additional resolution over species-specific cgMLST. Analyses of isolates from different common-source listeriosis outbreaks revealed various degrees of diversity, indicating that the numbers of allelic differences should always be combined with cgMLST clustering and epidemiological evidence to define a listeriosis outbreak.

**IMPORTANCE** Classic multilocus sequence typing (MLST) schemes targeting internal fragments of 6 to 8 genes that define clonal complexes or epidemic clones have been widely employed to study L. monocytogenes biodiversity and its relation to pathogenicity potential and epidemiology. We demonstrated that core genome MLST schemes can be used for the simultaneous identification of clonal groups and the differentiation of individual outbreak strains and epidemiologically unrelated strains of the same clonal group. We further developed lineage-specific cgMLST schemes that targeted more genomic regions than the species-specific cgMLST schemes. Our data revealed the genome-level diversity of clonal groups defined by classic MLST schemes. Our identification of U.S. and international outbreaks caused by major clonal groups can contribute to further understanding of the global epidemiology of L. monocytogenes.

## INTRODUCTION

Listeria monocytogenes is a Gram-positive, facultative, intracellular, foodborne pathogen that causes the disease listeriosis, which can be fatal, particularly in high-risk populations (1). L. monocytogenes had previously consisted of three major evolutionary lineages, I, II, and III (1). Recently. lineage IIIB, a subpopulation of lineage III, was redefined as lineage IV, as genomic comparisons showed that lineage IIIB was distinct enough from lineages IIIA and IIIB to be considered its own lineage (2). Among the 13 serotypes of L. monocytogenes, isolates of serotypes 4b, 1/2a, and 1/2b have been associated with the vast majority of listeriosis outbreaks and sporadic cases (3). It has been demonstrated that many different listeriosis outbreak strains, including those from different countries or continents, were genetically close and thus belonged to specific clonal groups (3).

The identification of these globally distributed clonal groups has been instrumental for assessing the biodiversity of L. monocytogenes and understanding the pathogenic potential and epidemiology related to different subpopulations of L. monocytogenes. Two nomenclature systems have been used to describe these clonal groups. One system is the epidemic clone (EC) system, in which an EC is defined as a clonal group that has been associated with more than one outbreak (3). ECs of L. monocytogenes were initially defined on the basis of the genotyping tools available (3) and subsequently defined on the basis of identical virulence types (VTs) of a 6-gene multi-virulence-locus sequence tying (MVLST) scheme (4–6); most recently, whole-genome sequencing (WGS) analysis using kSNP v2 software (hereinafter referred to as kSNP-WGS) was employed to define a new EC (7). The other nomenclature system is the clonal complex (CC) system, in which a CC is defined by a 7-gene multilocus sequence tying (MLST) scheme (1, 8) as a group of sequence types (STs) differing by no more than one allele from at least one other ST in the group, regardless of its involvement in outbreaks (9). Under the latter nomenclature system, a clonal group having an ST that differs from all other existing STs by at least two alleles is defined as a singleton (9). An 8-gene MvLST scheme (10), derived from the 6-gene MVLST, and the kSNP-WGS approach (7) both demonstrated that several previously identified ECs corresponded to CCs involved in more than one outbreak. Thus, in this article, we use the two nomenclature terms interchangeably for clonal groups involved in more than one outbreak (hereinafter referred to as CC/EC) and use CC only for clonal groups involved in no outbreaks or only one outbreak.

Although classic MLST schemes targeting internal fragments of 6 to 8 genes and a few other molecular subtyping methods could identify these clonal groups, none of the classic MLST schemes were able to differentiate isolates in an outbreak from epidemiologically unrelated isolates of the same clonal group (4, 10). In the study using kSNP-WGS analysis, most outbreaks were represented by only one isolate (7), so we could not determine whether isolates from different outbreaks could be distinctly clustered. Additional resolution power is needed for identifying both clonal groups and outbreak strains of L. monocytogenes, and the WGS technology can be used to provide that power. An outbreak strain was defined as a group of isolates that were both epidemiologically related and genetically related and thus presumed to be clonally related (11). Such a group of isolates was alternatively named an outbreak clone (6, 12), but we use the term outbreak strain throughout this article. Quite a few listeriosis outbreaks have involved multiple outbreak strains (7).

The availability of whole-genome sequences of L. monocytogenes allowed Ruppitsch et al. (12) to develop a core genome MLST (cgMLST) scheme targeting the entire species of L. monocytogenes, using 1,701 full-length core genes defined from 36 complete genomes and 6 draft genomes (hereinafter referred to as 1,701-cgMLST). However, many more genomes, especially complete genomes, from different lineages have become available in the National Center for Biotechnology Information (NCBI) archive since publication of that article, raising the question of whether the use of different sets of genomes to define core genes might yield a cgMLST scheme that produces a different phylogeny for the same set of isolates.

Isolates from different lineages of L. monocytogenes have exhibited vast genetic diversity (3), and therefore, a core gene set defined for the entire species of L. monocytogenes might not allow full utilization of the high resolution provided by WGS. For example, 1,701-cgMLST used a lineage II strain (EGD-e) as the reference genome to define core genes, which excluded some of the core genes and many accessory genes specific to lineage I and III isolates (12). In addition, it remains to be determined whether cgMLST can be used to identify major CCs or ECs and to differentiate different outbreak strains of the same CC or EC.

Our objectives for this study were to (i) investigate whether using different sets of genomes to define core genes would affect the usefulness of the resulting cgMLST schemes for identifying major lineages, serotypes, CCs, ECs, and outbreak strains, (ii) develop new cgMLST schemes specific for each of the major lineages of L. monocytogenes, (iii) determine whether lineage-specific schemes offer improved discriminatory power and epidemiological concordance over species-specific cgMLST schemes, (iv) evaluate whether the clusters identified by cgMLST are consistent with the clonal groups defined by the 7-gene MLST, and (v) evaluate whether cgMLST schemes can differentiate among epidemiologically unrelated strains of the same clonal group.

## MATERIALS AND METHODS

### Whole-genome sequences.

A total of 51 L. monocytogenes isolates representing 3 lineages, 6 CCs that are also ECs, an additional 16 CCs or singletons, and 9 outbreak strains from 9 outbreaks were obtained from the Center for Food Safety and Applied Nutrition (CFSAN) culture collection and were sequenced on an Illumina HiSeq 2000 platform (100-bp, paired-end reads; Illumina, Inc., San Diego, CA) using a Kapa HTP library preparation kit (catalog no. KK8234; Kapa Biosystems, Inc., Wilmington, MA) per the manufacturer's instructions (13). The genomic sequence contigs for each isolate were *de novo* assembled using Qiagen CLC Genomics Workbench software (version 8.0; Aarhus, Denmark). We then obtained all 68 complete genomes available at GenBank as of February 2016 and an additional 139 draft genomes that (i) met the quality criterion assessed as described below, (ii) complemented other genomes for species representation, and (iii) included well-identified outbreak-associated isolates (Table 1). We performed *in silico* MLST and MVLST to determine their lineage information and performed *in silico* PCR serogrouping using the tool built in the Pasteur MLST L. monocytogenes database (http://bigsdb.web.pasteur.fr/listeria/listeria.html) for isolates suspected to be serotype 4b variants. The combination of 51 genomes sequenced in this study and 207 genomes obtained from NCBI and the European Nucleotide Archive (ENA) represented three lineages (including lineage IIIB, which was redefined as lineage IV), 12 serotypes, 10 CCs that are also ECs, and an additional 43 CCs or singletons. A well-identified strain from a sporadic case, a well-identified strain from a recall, and 48 outbreak strains from 39 listeriosis outbreaks were represented. Among them, 33 CCs or ECs were represented by more than one isolate, and 29 strains from 24 outbreaks/incidents were represented by more than one isolate. These 258 genomes included the 42 genomes used to define the 1,701-cgMLST (12) and 23 of the genomes reported by Maury et al. (14) because those genomes complemented other genomes for species representation.

**TABLE 1:** Information on all isolates analyzed in this study, sorted in the order of CC designations

Isolate identifier/alternative identifier^*i*^	Serotype/lineage	Metadata	ST^*a*^	CC^*b*^	EC^*c*^	NCBI or ENA accession no. or Sequence Read Archive identifier
NE dc2014*	I	Food	1	1	I	NZ_CP007492.1
WSLC1042*/ATCC 23074*	4b/I	NA^*d*^	79	1	I	NZ_CP007210.1
WSLC1018*	4e/I	Pre-1961 UK, poultry	73	1	I	NZ_CP013285.1
SLCC2378*/ATCC 19118*	4e/I	Chicken	73	1	I	NC_018585.1
NTSN*	4b/I	2011 China, sheep	1	1	I	NZ_CP009897.1
BL9302	4b/I	1987 Pennsylvania outbreak, clinical	1	1	I	SRR1814376
SLCC208^§^	4b/I	1921 France, clinical	495	1	I	LMXJ00000000.1
J1-112	4b/I	1976 France Anjou outbreak, clinical	1	1	I	SRR1814389
LL195*	4b/I	1983–1987 Switzerland cheese outbreak, clinical	1	1	I	NC_019556.1
J1-123	4b/I	1983–1987 Switzerland cheese outbreak, clinical	1	1	I	SRR1814365
F2365*/J1-110*	4b/I	1985 California cheese outbreak, clinical	1	1	I	NC_002973.6
J1-119	4b/I	1985 California cheese outbreak, clinical	1	1	I	SRR1814334
J1-108*	4b/I	1981 Canada coleslaw outbreak, clinical	1	1	I	NC_021825.1
J1-003	4b/I	1981 Canada coleslaw outbreak, clinical	1	1	I	SRR1814363
N3-008	4b/I	1981 Canada coleslaw outbreak, food	1	1	I	SRR3707731
W9708^§^	4b/I	2008 Austria jellied pork outbreak, food	1	1	I	ERR664384
W9508^§^	4b/I	2008 Austria jellied pork outbreak, food	1	1	I	ERR664382
L4508^§^	4b/I	2008 Austria jellied pork outbreak, clinical	1	1	I	ERR664379
L3908^§^	4b/I	2008 Austria jellied pork outbreak, clinical	1	1	I	ERR664377
CFSAN028555^§^	4b/I	2014–2015 U.S. multistate caramel apple outbreak, food	1	1	I	SRR1763864
CFSAN028558^§^	4b/I	2014–2015 U.S. multistate caramel apple outbreak, food	1	1	I	SRR1763833
CFSAN028872^§^	4b/I	2014–2015 U.S. multistate caramel apple outbreak, food	1	1	I	SRR1770498
CFSAN028879^§^	4b/I	2014–2015 U.S. multistate caramel apple outbreak, food	1	1	I	SRR1770500
PNUSAL001157^§^	4b/I	2014–2015 U.S. multistate caramel apple outbreak, clinical	1	1	I	LNSL00000000.1
LM05-00008^§^	I	France, clinical	1	1	I	CYUP00000000.1
LM05-00172^§^	I	France, clinical	1	1	I	CYUS00000000.1
LM07-00596^§^	I	France, clinical	1	1	I	CYVI00000000.1
R2-578	4b/I	1983 Boston, MA, milk outbreak, clinical	2	2	IV	SRR1814368
R2-583	4b/I	1983 Boston, MA, milk outbreak, clinical	2	2	IV	SRR3707880
ScottA^§^	4b/I	1983 Boston, MA, milk outbreak, clinical	290	2	IV	CM001159.1
LI0521^§^	4b/I	1983 Boston, MA, cheese outbreak	2	2	IV	JMMW00000000.1
J1-220*	4b/I	1979 Boston, MA, vegetable outbreak	2	2	IV	NC_021829.2
WSLC1047*	4d/I	NA	290	2	IV	NZ_CP013289.1
ATCC 19117*	4d/I	Pre-1961 United States, sheep	2	2	IV	NC_018584.1
J1-129	4b/I	1987–1989 UK pâté outbreak, clinical	48	2	IV	SRR1814364
J1-116	4b/I	1987–1989 UK pâté outbreak, clinical	257	2	IV	SRR1814378
N3-013	4b/I	1987–1989 UK pâté outbreak, food	2	2	IV	SRR3707715
N3-010	4b/I	1987–1989 UK pâté outbreak, food	2	2	IV	SRR3707727
Lm_1824^§^	4b/I	2012 Italy, cheese facility	2	2	IV	AZIV00000000.1
HPB2262^§^	4b/I	1997 Italy corn gastroenteritis outbreak, clinical	2	2	IV	AATL00000000.2
LS267^§^	4b/I	1993–1994 Texas frozen vegetable outbreak, clinical	2	2	IV	LNNM00000000.1
LS268^§^	4b/I	1993–1994 Texas frozen vegetable outbreak, clinical	2	2	IV	LNNN00000000.1
LS269^§^	4b/I	1993–1994 Texas frozen vegetable outbreak, clinical	2	2	IV	LNNO00000000.1
LS270^§^	4b/I	1993–1994 Texas frozen vegetable outbreak, clinical	2	2	IV	LNNP00000000.1
LS275^§^	4b/I	1993–1994 Texas frozen vegetable outbreak, clinical	2	2	IV	LNNS00000000.1
LS276^§^	4b/I	1993–1994 Texas frozen vegetable outbreak, clinical	2	2	IV	LNNT00000000.1
CFSAN023951^§^	I	2014 U.S. multistate cheese outbreak, environment	2	2	IV	SRR1619552
CFSAN023952^§^	I	2014 U.S. multistate cheese outbreak, environment	2	2	IV	SRR1635202
CFSAN025771^§^	I	2014 U.S. multistate cheese outbreak, food	NO^*e*^	2	IV	SRR1980624
CFSAN025772^§^	I	2014 U.S. multistate cheese outbreak, food	NO	2	IV	SRR1980616
47G^§^	4b/I	2006 Brazil, dairy product	2	2	IV	LKCY00000000.1
R2-502*	1/2b/I	1994 Illinois chocolate milk gastroenteritis outbreak	3	3	VIII	NC_021838.1
G6054^§^/R2-503^§^	1/2b/I	1994 Illinois chocolate milk gastroenteritis outbreak	3	3	VIII	JPTW00000000.1
BL0129	1/2b/I	1999 United States, food	3	3	VIII	SRR1814375
N1-011A*	1/2b/I	Environment	3	3	VIII	NC_021826.1
SLCC2755*	1/2b/I	1967, chinchilla	66	3	VIII	NC_018587.1
SLCC2482*	7/I	1966, clinical	3	3	VIII	NC_018591.1
F4233^§^	1/2b/I	1987 Pennsylvania outbreak, clinical	3	3	VIII	JMUA00000000.1
N1-017^§^	1/2b/I	1998, food	3	3	VIII	AARP00000000.4
LM09-00558^§^	I	France, clinical	4	4	X	CYWW00000000.1
CLIP 80459*	4b/I	1999–2000 France pork rillettes outbreak, clinical	4	4	X	NC_012488.1
07PF0776*	4b/I	Massachusetts sporadic case, clinical	4	4	X	NC_017728.1
L312*	4b/I	Cheese	4	4	X	NC_018642.1
N2306*	4b/I	2013–2014 Switzerland salad outbreak, clinical	4	4	X	NZ_CP011004.1
L1254^§^	1/2b/I	2009 United States, clinical	5	5	VI	JNFI00000000.1
Lm_1886^§^	1/2b/I	2012 Italy, cheese facility	5	5	VI	AZIX00000000.1
CFSAN000794^§^	1/2b/I	2011 U.S. multistate cantaloupe outbreak, food	5	5	VI	SRR1908945
CFSAN000779^§^	1/2b/I	2011 U.S. multistate cantaloupe outbreak, environment	5	5	VI	SRR1908952
L2624*	1/2b/I	2011 U.S. multistate cantaloupe outbreak, clinical	5	5	VI	NZ_CP007686.1
CFSAN023460^§^	1/2b/I	2014 U.S. stone fruit recall, food	5	5	VI	SRR1556287
CFSAN023476^§^	1/2b/I	2014 U.S. stone fruit recall, food	5	5	VI	SRR1553871
CFSAN024090^§^	1/2b/I	2014 U.S. stone fruit recall, environment	5	5	VI	SRR1571543
CFSAN023459*	1/2b/I	2014 U.S. stone fruit recall, food	5	5	VI	NZ_CP014252.1
CFSAN010068*	1/2b/I	2013 U.S. Hispanic-style cheese outbreak, food	5	5	VI	NZ_CP014250.1
CFSAN010085^§^	1/2b/I	2013 U.S. Hispanic-style cheese outbreak, food	5	5	VI	SRR1182223
CFSAN010087^§^	1/2b/I	2013 U.S. Hispanic-style cheese outbreak, food	5	5	VI	SRR1181522
CFSAN011016^§^	1/2b/I	2013 U.S. Hispanic-style cheese outbreak, food	5	5	VI	SRR1378358
CFSAN033566^§^	1/2b/I	2010–2015 U.S. multistate ice cream outbreak, food	5	5	VI	SRR2054281
CFSAN034262^§^	1/2b/I	2010–2015 U.S. multistate ice cream outbreak, food	5	5	VI	SRR2078889
CFSAN033576^§^	1/2b/I	2010–2015 U.S. multistate ice cream outbreak, food	5	5	VI	SRR2054168
CFSAN033577^§^	1/2b/I	2010–2015 U.S. multistate ice cream outbreak, food	5	5	VI	SRR2054250
CFSAN030677^§^	3b/I	2010–2015 U.S. multistate ice cream outbreak, food	5	5	VI	SRR1955840
CFSAN032211^§^	3b/I	2010–2015 U.S. multistate ice cream outbreak, food	5	5	VI	SRR1998952
CFSAN032223^§^	3b/I	2010–2015 U.S. multistate ice cream outbreak, food	5	5	VI	SRR1982133
CFSAN032213^§^	3b/I	2010–2015 U.S. multistate ice cream outbreak, food	5	5	VI	SRR1998966
L1181^§^	1/2b/I	2009 United States, clinical	5	5	VI	JNGR00000000.1
BL0102	3b/I	1996 United States, clinical	5	5	VI	SRR3707716
J2-064*	1/2b/I	1989 United States, cow	5	5	VI	NC_021824.1
CFSAN008100*	I	2014 United States, food	5	5	VI	NZ_CP011398.1
IZSAM_Lm_hs2008*	4b/I	2009 Italy, clinical	6	6	II	NZ_CP010346.1
H7355	4b/I	1998 U.S. multistate hot dog outbreak, clinical	6	6	II	SRR1814362
H7961	4b/I	1998 U.S. multistate hot dog outbreak, food	6	6	II	SRR1814399
H7969	4b/I	1998 U.S. multistate hot dog outbreak, clinical	6	6	II	SRR3707879
H7550	4b/I	1998 U.S. multistate hot dog outbreak, clinical	6	6	II	SRR1815437
H7557	4b/I	1998 U.S. multistate hot dog outbreak, food	6	6	II	SRR3707886
H7596	4b/I	1998 U.S. multistate hot dog outbreak, food	6	6	II	SRR1815440
H7738	4b/I	1998 U.S. multistate hot dog outbreak, food	6	6	II	SRR3707884
H7762	4b/I	1998 U.S. multistate hot dog outbreak, food	6	6	II	SRR3707885
H7962	4b/I	1998 U.S. multistate hot dog outbreak, food	6	6	II	SRR1815438
J1776*	4b/I	2002 U.S. multistate turkey deli meat outbreak, clinical	6	6	II	NC_021839.1
J1926*	4b/I	2002 U.S. multistate turkey deli meat outbreak, food	6	6	II	NC_021840.1
J1817*	4b/I	2002 U.S. multistate turkey deli meat outbreak, environment	6	6	II	NC_021827.1
J1816*	4b/I	2002 U.S. multistate turkey deli meat outbreak, environment	6	6	II	NC_021830.2
J1703	4b/I	2002 U.S. multistate turkey deli meat outbreak, clinical	6	6	II	SRR3707894
J1705	4b/I	2002 U.S. multistate turkey deli meat outbreak, clinical	6	6	II	SRR3707893
J1736	4b/I	2002 U.S. multistate turkey deli meat outbreak, clinical	6	6	II	SRR1815439
J1735	4b/I	2002 U.S. multistate turkey deli meat outbreak, clinical	6	6	II	SRR3707726
J1815	4b/I	2002 U.S. multistate turkey deli meat outbreak, environment	6	6	II	SRR3707728
J1925	4b/I	2002 U.S. multistate turkey deli meat outbreak, food	6	6	II	SRR1814333
J1927	4b/I	2002 U.S. multistate turkey deli meat outbreak, food	6	6	II	SRR3707892
CFSAN006121^§^	4b/I	2013 U.S. multistate French-style cheese outbreak, food	6	6	II	SRR955385
CFSAN006123^§^	4b/I	2013 U.S. multistate French-style cheese outbreak, food	6	6	II	SRR955387
CFSAN006122*	4b/I	2013 U.S. multistate French-style cheese outbreak, food	6	6	II	NZ_CP007600.1
BL9502	1/2a/II	1981 UK, clinical	7	7	VII	SRR1814366
J2692^§^	1/2a/II	2003 United States, clinical	7	7	VII	JNGJ00000000.1
L2626*	1/2a/II	2011 U.S. multistate cantaloupe outbreak, clinical	561	7	VII	NZ_CP007684.1
L2676*	1/2a/II	2011 U.S. multistate cantaloupe outbreak, clinical	7	7	VII	NZ_CP007685.1
LIS0087^§^	1/2a/II	2011 U.S. multistate cantaloupe outbreak, environmental	7	7	VII	SRR3644931
LIS0077^§^	1/2a/II	2011 U.S. multistate cantaloupe outbreak, environmental	561	7	VII	SRR3644928
SLCC5850*	1/2a/II	1924 UK, rabbit	12	7	VII	NC_018592.1
EGD*	1/2a/II	1924 UK, rabbit	12	7	VII	NC_022568.1
WSLC1001*	1/2a/II	1935 UK, clinical	12	7	VII	NZ_CP007160.1
2015TE19005-1355*	II	2014–2016 Italy outbreak	7	7	VII	NZ_CP014261.1
L1846*	1/2a/II	2010 Louisiana head cheese outbreak, clinical	7	7	VII	NZ_CP007688.1
10403S*	1/2a/II	Pre-1968, derivative of 10403 from U.S. clinical case	85	7	VII	NC_017544.1
HPB5415^§^	1/2a/II	2008 Canada deli meat outbreak, food	292	8	V	JOKV00000000.1
08-5578*	1/2a/II	2008 Canada deli meat outbreak, clinical	292	8	V	NC_013766.2
08-5923*	1/2a/II	2008 Canada deli meat outbreak, clinical	120	8	V	NC_013768.1
Lm60*	1/2a/II	2006 Switzerland, clinical	551	8	V	NZ_CP009258.1
R479a*	1/2a/II	1996–1999 smoked salmon, persistent isolate	8	8	V	NZ_HG813247.1
Lm N1546*	1/2a/II	2011 Switzerland ham outbreak, clinical	8	8	V	NZ_CP013724.1
IZSAM_Lm15_17439*	1/2a/II	2008 Italy outbreak, clinical	8	8	V	NZ_CP013919.1
Lm_1823^§^	1/2a/II	2012 Italy, cheese facility	8	8	V	AZIU00000000.1
Lm_1889^§^	1/2a/II	2012 Italy, cheese facility	8	8	V	AZIY00000000.1
55G^§^	1/2a/II	2006 Brazil, dairy	9	9		LKHO00000000.1
BL0137	1/2c/II	2000 United States, food	9	9		SRR1814383
BL0101	1/2c/II	UK sporadic case, clinical	9	9		SRR3707717
LM57179^§^	1/2c/II	France, clinical	9	9		CYUW01000020.1
LM6186^§^	1/2c/II	France, clinical	9	9		CYVG01000006.1
LM77097^§^	1/2c/II	France, clinical	9	9		CYWC01000055.1
LM80661^§^	1/2c/II	France, clinical	9	9		CYWL01000016.1
SLCC2372*	1/2c/II	1935 UK, clinical	122	9		NC_018588.1
SLCC2479*	3c/II	1966	9	9		NC_018589.1
EGD-e*	1/2a/II	1924 UK, rabbit	35	9		NC_003210.1
FSL R2-561*	1/2c/II	UK, clinical	9	9		NC_017546.1
J0221^§^	1/2a/II	2000 U.S. sporadic case, clinical	11	11	III	JNGL00000000.1
J0161*	1/2a/II	2000 U.S. multistate turkey deli meat outbreak, clinical	11	11	III	NC_017545.1
J2818^§^	1/2a/II	2000 U.S. multistate turkey deli meat outbreak, food	11	11	III	AARX00000000.2
F6900^§^	1/2a/II	1988 U.S. sporadic case, clinical	11	11	III	AARU00000000.2
F6854^§^/J1-101^§^	1/2a/II	1988 U.S. sporadic case, food	11	11	III	NZ_AADQ00000000.1
L1023^§^	1/2a/II	2009 U.S. multistate cheese outbreak, clinical	NO	11	III	JNGN00000000.1
F4235^§^	1/2a/II	1987 Pennsylvania outbreak, clinical	11	11	III	JNGK00000000.1
J0847^§^	1/2a/II	2001 United States, clinical	11	11	III	JNGM00000000.1
SLCC7179*	3a/II	1986 Austria, cheese	91	14		NC_018593.1
SLCC0759^§^	1/2a/II	NA	481	18		ERR664779
SLCC1042^§^	1/2a/II	NA	18	18		ERR664780
Lm 3136*	1/2a/II	2005 Switzerland cheese outbreak, clinical	18	18		NZ_CP013723.1
LM83088^§^	1/2a/II	France, clinical	19	19		CYWM01000033.1
L2074*	1/2a/II	2010 Texas celery outbreak, clinical	378	19		NZ_CP007689.1
LM07-00514^§^	II	Clinical	26	26		CYVH00000000.1
Lm 3163*	1/2a/II	2005 Switzerland cheese outbreak, clinical	26	26		NZ_CP013722.1
L2625*	1/2a/II	2011 U.S. multistate cantaloupe outbreak, clinical	29	29		NZ_CP007687.1
BL0108	1/2a/II	1994 United States, animal	29	29		SRR3707865
SLCC3287^§^	1/2a/II	NA	427	29		ERR664782
LM07421^§^	II	Food	31	31		CYWA00000000.1
LM07422^§^	II	Food	31	31		CYWB00000000.1
F2-032	1/2a/II	1999 United States, food	31	31		SRR1814387
CFSAN007956*	II	NA	37	37		NZ_CP011397.1
LM09-01099^§^	II	France, clinical	37	37		CYXB00000000.1
LM07-01337^§^	I	France, clinical	54	54		CYVO01000001.1
G4599^§^	1/2b/I	1993 Italy rice salad gastroenteritis outbreak	59	59		JPTX00000000.1
DPC6895^§^	1/2b/I	Raw cow milk	59	59		LABG00000000.1
BL0122/X1-002	4a/IIIA	Europe, food	201	69		SRR1814352
HCC23*	4a/IIIA	United States, catfish	201	69		NC_011660.1
L99*	4a/IIIA	1950 Netherlands, cheese	201	69		NC_017529.1
M7*	4a/IIIA	China, cow milk	201	69		NC_017537.1
LM850658*	4a/IIIA	1985 Australia, sheep	202	69		NZ_CP009242.1
BL0112	I	United States, food	87	87		SRR1814400
J1-194^§^	1/2b/I	1997 New York, clinical	88	88		AARJ00000000.2
J2-003	1/2a/II	1993 United States, animal	391	89		SRR1814363
LM06-00983^§^	II	France, clinical	391	89		CYVB00000000.1
N3-165^§^	1/2a/II	2002 New York, environment	222	90		AARQ00000000.2
F3-831	II	2004 United States, environment	222	90		SRR1814340
2012-L5240^§^	1/2a/II	2012 U.S. multistate ricotta cheese outbreak, clinical	101	101		JNGO00000000.1
2012-L5322^§^	1/2a/II	2012 U.S. multistate ricotta cheese outbreak, food	101	101		JNGP00000000.1
2012-L5323^§^	1/2a/II	2012 U.S. multistate ricotta cheese outbreak, clinical	101	101		JNGY00000000.1
2012-L5324^§^	1/2a/II	2012 U.S. multistate ricotta cheese outbreak, food	101	101		JNGZ00000000.1
Lm_1840^§^	1/2a/II	2012 Italy, cheese facility	101	101		AZIW00000000.1
NCCP No. 15743^§^	1/2a/II	2012 South Korea, stool	101	101		APJT00000000.1
Lm_1880^§^	1/2a/II	2012 Italy, cheese facility	121	121		AZIZ00000000.1
6179*	1/2a/II	Ireland cheese facility, persistent strain	121	121		NZ_HG813249.1
BL0202	1/2a/II	United States, food	121	121		SRR3707878
JF5171^§^	1/2a/II	2006 Switzerland, bovine	124	ST124^*f*^		JWLP01000000.1
SLCC2376*/ATCC 19116*	4c/IIIA	Pre-1961 England, chicken	71	131		NC_018590.1
WSLC1019*	4c/IIIA	Pre-1961 England, chicken	71	131		NZ_CP013286.1
ATCC 19114*	4a/IIIA	1931 United States or New Zealand animal	71	131		NZ_CP013287.1
SLCC85^§^	IIIA	NA	623	131		CYXE01000009.1
Finland 1998*	3a/II	1998 Finland butter outbreak	155	155		NC_017547.1
C1-387*	1/2a/II	1999 New York, meat	155	155		NC_021823.1
LM07424^§^	1/2a/II	Food	193	193		CYVZ00000000.1
LM07425^§^	1/2a/II	Food	193	193		CYWO00000000.1
LM07456^§^	1/2a/II	Cheese	196	193		CYWE00000000.1
LM06-01614^§^	II	Colombia, environment	199	199		CYVF00000000.1
BL0120	1/2a/II	1998 United States, clinical	199	199		SRR3707729
BL0106/J1-168	4a/IIIA	1996 U.S. sporadic case, clinical	203	203		SRR1814374
LM07-01067^§^	I	France, clinical	386	224		CYVM00000000.1
BL0133	I	2002 United States, environment	224	224		SRR3707876
LS643^§^	4bv/I	Australia, clinical	240	240		AVQN00000000.1
LS644^§^	4bv/I	Australia, clinical	240	240		AVQO00000000.1
LS645^§^	4bv/I	Australia, clinical	240	240		AVQP00000000.1
LM10-00505^§^	I	France, clinical	194	315		CYUN00000000.1
SLCC2540*	3b/I	1956 United States, clinical	617	344		NC_018586.1
LS642^§^	4bv/I	Australia, clinical	572	373		AVQM00000000.1
CFSAN027922^§^	4bv/I	2014–2015 U.S. multistate caramel apple outbreak, environment	382	ST382	IX	SRR1767799
CFSAN027923^§^	4bv/I	2014–2015 U.S. multistate caramel apple outbreak, environment	382	ST382	IX	SRR1767800
CFSAN028556^§^	4bv/I	2014–2015 U.S. multistate caramel apple outbreak, food	382	ST382	IX	SRR1763846
CFSAN028559^§^	4bv/I	2014–2015 U.S. multistate caramel apple outbreak, food	382	ST382	IX	SRR1763839
PNUSAL001166^§^	4bv/I	2014–2015 U.S. multistate caramel apple outbreak, clinical	382	ST382	IX	LNSM00000000.1
PNUSAL001024^§^	4bv/I	2014 U.S. multistate stone fruit outbreak, clinical	382	ST382	IX	LNSO00000000.1
CFSAN023463*	4bv/I	2014 U.S. multistate stone fruit outbreak, food	382	ST382	IX	NZ_CP012021.1
CFSAN023473^§^	4bv/I	2014 U.S. multistate stone fruit outbreak, food	382	ST382	IX	SRR1553779
CFSAN023474^§^	4bv/I	2014 U.S. multistate stone fruit outbreak, food	382	ST382	IX	SRR1553906
CFSAN023491^§^	4bv/I	2014 U.S. multistate stone fruit outbreak, food	382	ST382	IX	SRR1553882
CFSAN024077^§^	4bv/I	2014 U.S. multistate stone fruit outbreak, environment	382	ST382	IX	SRR1571519
PNUSAL000870^§^	4bv/I	2014 U.S. multistate stone fruit outbreak, clinical	382	ST382	IX	LNSP00000000.1
CFSAN045511^§^	4bv/I	2015–2016 U.S. multistate packaged salad outbreak, food	382	ST382	IX	SRR3147176
CFSAN045513^§^	4bv/I	2015–2016 U.S. multistate packaged salad outbreak, food	382	ST382	IX	SRR3147177
CFSAN045515^§^	4bv/I	2015–2016 U.S. multistate packaged salad outbreak, food	382	ST382	IX	SRR3147179
CFSAN045517^§^	4bv/I	2015–2016 U.S. multistate packaged salad outbreak, food	382	ST382	IX	SRR3147180
CFSAN003417^§^	4bv/I	United States, monkey	382	ST382	IX	SRR1818112
BL0104/J1-031	4a/IIIA	Canada, clinical	557	396		SRR1814370
3230TP3^§^	1/2a/II	2009–2010 Austria/Germany acid curd cheese outbreak, food	398	398		ERR538094
4548TP4^§^	1/2a/II	2009–2010 Austria/Germany acid curd cheese outbreak, food	398	398		ERR538096
L10-10^§^	1/2a/II	2009–2010 Austria/Germany acid curd cheese outbreak, clinical	398	398		ERR538115
L14-10^§^	1/2a/II	2009–2010 Austria/Germany acid curd cheese outbreak, clinical	398	398		ERR538116
QOC2^§^	1/2a/II	2009–2010 Austria/Germany acid curd cheese outbreak, clinical	398	398		CBVW000000000.1
QOC1^§^	1/2a/II	2009–2010 Austria/Germany acid curd cheese outbreak, clinical	403	403		CBVZ000000000.1
3230TP5^§^	1/2a/II	2009–2010 Austria/Germany acid curd cheese outbreak, food	403	403		ERR538095
L21-09^§^	1/2a/II	2009–2010 Austria/Germany acid curd cheese outbreak, clinical	403	403		ERR538124
L23-09^§^	1/2a/II	2009–2010 Austria/Germany acid curd cheese outbreak, clinical	403	403		ERR538125
L29-09^§^	1/2a/II	2009–2010 Austria/Germany acid curd cheese outbreak, clinical	403	403		ERR538127
L31-09^§^	1/2a/II	2009–2010 Austria/Germany acid curd cheese outbreak, clinical	777	403		ERR538129
LM07-01345^§^	II	France, clinical	620	412		CYVP00000000.1
BL0138	II	2002 United States, environment	412	412		SRR1814388
J2-031*	1/2a/II	1996 New York, animal	394	415		NC_021837.1
SLCC6263^§^	1/2a/II	NA	466	ST466		ERR664785
SLCC4771^§^	4c/IIIB	NA	467	ST467		ERR664786
SLCC0717^§^	1/2a/II	NA	518	ST518		ERR664778
LS542^§^	4bv/I	United States, environment	554	554		AVQQ00000000.1
W1-111^§^	4c/IIIB	United States	562	ST562		SRR3707730
J1-208^§^	4a/IIIB	1998 United States, animal	569	ST569		AARL00000000.2
L1118^§^	1/2a/II	2009 U.S. multistate sprout outbreak, clinical	573	ST573		JNGQ00000000.1
BL0103	4b/I	United States, clinical	801	651		SRR3707734
H1-030	II	2000 United States, environment	635	635^*g*^		SRR1814380
H1-051	II	2000 United States, environment	NO	635		SRR1814384
BL0109	4b/I	1989 United States, animal	663	ST663		SRR3707866
BL0105/W1-110	4c/IIIC	NA	NO	NS^*h*^		SRR1814377
BL0142/F2-525	4b/IIIA	2000 U.S. sporadic case, clinical	NO	NS		SRR1814386

a ST, sequence type.

b CC, clonal complex.

c EC, epidemic clone.

d NA, metadata not available.

e NO, STs were not observed in the Pasteur MLST database. If an ST differed from an existing ST by one allele, it was assigned to a CC.

f Numbers starting with ST indicate singletons.

g We tentatively named this clonal group CC635 because H1-030 belonged to singleton ST635 and H1-051 differed from H1-030 by one MLST allele.

h NS, STs were not observed in the Pasteur MLST database and differed from any existing ST by at least two alleles, and thus, these could not be assigned to any CC.

i *, complete genomes obtained from NCBI; §, draft genomes obtained from NCBI and ENA. Isolates not labeled * or § were sequenced in this study.

### Development of cgMLST schemes.

Out of the 68 complete genomes, 67 were used for the development of cgMLST schemes as well as the evaluation of cgMLST for clonal group and outbreak strain identification (Table 1). The other genome (SLCC2482) was annotated as a complete genome but contained an unclosed gap (15) and thus was not used to define core genes. These genomes represented lineages I, II, and III (including lineage IIIB, which was redefined as lineage IV) and 11 serotypes. Compared to the genomes used to define the 1,701-cgMLST scheme (12), all 6 draft genomes were removed and 31 additional complete genomes were added. In addition, the reference genome was that of the same strain (EGD-e) (GenBank accession no. NC_003210.1, dated December 2015) (16). This resulted in a species-specific scheme, the L. monocytogenes cgMLST (Lm-cgMLST) scheme containing 1,827 core genes (hereinafter referred to as 1,827-cgMLST). We further developed three lineage-specific schemes. To develop an L. monocytogenes lineage I-specific scheme (LmLI-cgMLST), 31 complete genomes were used with J1776 (GenBank accession no. NC_021839.1, dated August 2015) (17) as the reference. To develop an L. monocytogenes lineage II-specific scheme (LmLII-cgMLST), 29 complete genomes were used with EGD-e (GenBank accession no. NC_003210.1, dated December 2015) as the reference. To develop an L. monocytogenes lineage III-specific scheme (LmLIII-cgMLST), 7 complete genomes were used with HCC23 (GenBank accession no. NC_011660.1, dated July 2015) (18) as the reference. Due to the limited number of lineage IV isolates for which the WGS was available, we used the original lineage III definition that included lineage IIIB. Genome-wide gene-by-gene comparisons were performed using the cgMLST target definer (version 3.1.0) function of SeqSphere^+^ software (Ridom GmbH, Germany) with default parameters, as described by Ruppitsch et al. (12).

### Evaluation of cgMLST schemes for identification of clonal groups and differentiation of outbreak strains from epidemiologically unrelated strains of the same clonal group.

The 51 draft genomes sequenced in this study were used to evaluate the target gene representativeness for each cgMLST scheme. A cgMLST target gene set was considered to be representative if any L. monocytogenes genome contained ≥95% of the target genes determined by SeqSphere^+^ software (Ridom GmbH) (12). Subsequent cgMLST analyses were performed on the 51 draft genomes sequenced in this study, 68 complete genomes from the NCBI archive, and 139 additionally chosen draft genomes from NCBI or ENA that had >95% of the cgMLST genes, except for the genomes of strains F6854 (19), SLCC4771 (12), and J1-208 (2), which contained 91% to 93% of the genes. These were included because F6854 was one of only two isolates associated with a well-studied 1988 U.S. clinical case (19) for which WGS data were available; SLCC4771 and J1-208 were two of the small panel of lineage III isolates for which WGS data were available. The genes were extracted from these isolates using the default parameters in SeqSphere^+^ software (Ridom GmbH) as described by Ruppitsch et al. (12). Alleles for each gene were assigned automatically by the SeqSphere^+^ software (Ridom GmbH), and the combination of all alleles in each isolate formed an allelic profile that was used to generate neighbor-joining (NJ) trees using the parameter “pairwise ignore missing values” during distance calculation. We then calculated the maximum number of pairwise allelic differences among isolates of each CC or EC and isolates of each outbreak strain.

We first generated trees for the entire set of isolates using 1,701-cgMLST and 1,827-cgMLST to investigate whether the use of a slightly different set of core genes would yield different results in the identification of CC, EC, and outbreak strains. We then applied lineage-specific cgMLST schemes with the option to include accessory genes to evaluate whether these schemes could improve the performance for the identification of CC, EC, and outbreak strains over that of 1,701-cgMLST or 1,827-cgMLST.

*In silico* MLST and MVLST were performed on the isolates using the tools in SeqSphere^+^ software (Ridom GmbH). CCs were then assigned on the basis of the definition of Ragon et al. (9) and in the Pasteur MLST database (http://bigsdb.pasteur.fr/listeria/listeria.html); ECs were assigned as CCs involved in more than one outbreak and based on previous designations (3–7). The numbers of MLST STs and MVLST VTs in each clonal group were also compared.

### Accession number(s).

The NCBI Sequence Read Archive identifiers of the isolates sequenced in this study are provided in Table 1 (see footnote *i* of Table 1 for the key to the identification of isolates sequenced in this study).

## RESULTS AND DISCUSSION

### Core genome MLST (cgMLST) schemes with slightly different gene sets generated a generally congruent clustering of major lineages, serogroups, clonal groups, and outbreak strains.

**FIG 1:**
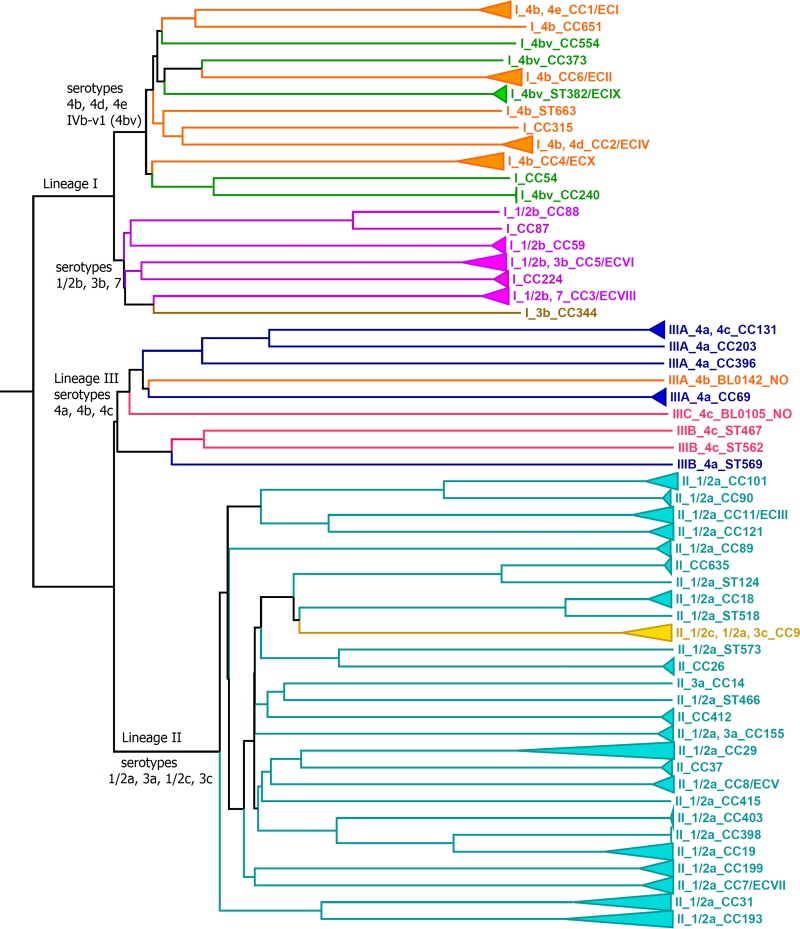
Phylogenetic tree of all isolates determined by 1,827-cgMLST. Clonal complexes (CCs) or epidemic clones (ECs) were defined by MLST. CC or EC clades containing more than one isolate are collapsed into triangles. The lineage, serotype (if available), CC, singleton, or EC names are listed in the taxa for all except two isolates (BL0142 and BL0105), for which the sequence types were not observed in the Pasteur MLST database and were thus designated NO. CCs or ECs of the same serotype have the branches and taxa in the same color. Serotype IVb-v1 (4bv) is a serotype 4b variant and has a color different from the color used for serotype 4b. If a CC or EC contains more than one serotype, the color representing the serotype of the majority of the isolates is used. If a CC or EC does not have serotype information available, the color of the nearest branch is used. None of the triangles contain isolates from more than one CC or EC. The triangle length is proportional to the diversity of isolates within each clonal group. CC398 isolates and CC240 isolates had relatively low levels of diversity, and thus, their triangles appear as vertical lines.

The 51 genomes sequenced in this study had coverage of 45 to 160 times, and ≥98% genes were targeted by our Lm-cgMLST scheme (referred to as the 1,827-cgMLST in this article) (see Table S1 in the supplemental material), which met the criterion (containing ≥95% of genes) of cgMLST target gene representativeness (12). Analyses of the 258 genomes (51 from this study and 207 from NCBI or ENA) revealed that species-specific cgMLST (1,701- and 1,827-cgMLST) clusters correlated with lineages, PCR serogroups (20), epidemic clones (ECs), clonal complexes (CCs), and outbreak strains (Fig. 1; see also Fig. S1 and S2 in the supplemental material). The serogroup consisting of serotypes 4b, 4d, and 4e and the serogroup comprising serotypes 1/2b, 3b, and 7 formed two distinct clades in the lineage I cluster. Isolates of serotype IVb-v1 (a serotype 4b variant alternatively named 4bv) were distributed among four clades/branches: CC554, CC373, singleton ST382/epidemic clone IX (ECIX), and CC240. The serogroup consisting of serotypes 1/2c and 3c formed a clade in CC9 in the lineage II cluster. The serogroup comprising serotypes 4a and 4c and a rare lineage III serotype 4b isolate (BL0142) formed a cluster representing lineage III. The three lineage IIIB isolates formed a clade that was separated from lineage IIIA and IIIC isolates. These data also confirmed a previous suggestion that ECIa be renamed ECIV (4): ECI and ECIV belonged to two distinct lineage I clades.

We developed another cgMLST scheme using 1,527 core genes (hereinafter referred to as 1,527-cgMLST), which were defined by adding some random draft genomes taken from NCBI. The 1,527-cgMLST scheme still generated clusters congruent with major clonal groups and outbreak strains (data not shown). This indicates that for general identification of the clonal groups and outbreak strains, precise selection of core genes may not be critical, and it is possible that the minimal number of genes necessary for this purpose might be less than what we have employed in this study. Despite this, isolates from three outbreaks/incidents could not be accurately clustered by either 1,827-cgMLST, 1,701-cgMLST, or 1,527-cgMLST, as described below. This led us to devise lineage-specific cgMLST schemes to determine whether discriminatory power could be improved.

### Species- and lineage-specific cgMLST identified clonal groups and distinguished outbreak strains from epidemiologically unrelated strains of the same clonal group.

**FIG 2:**
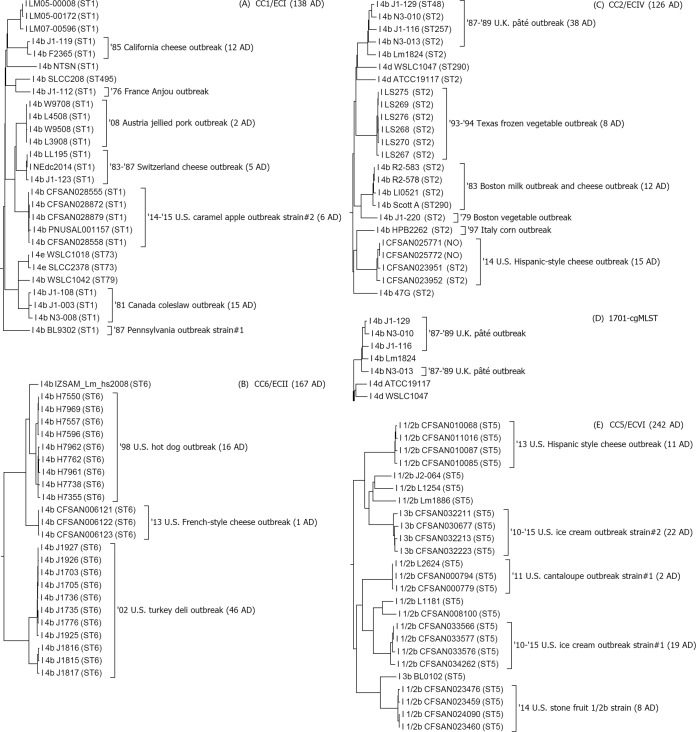

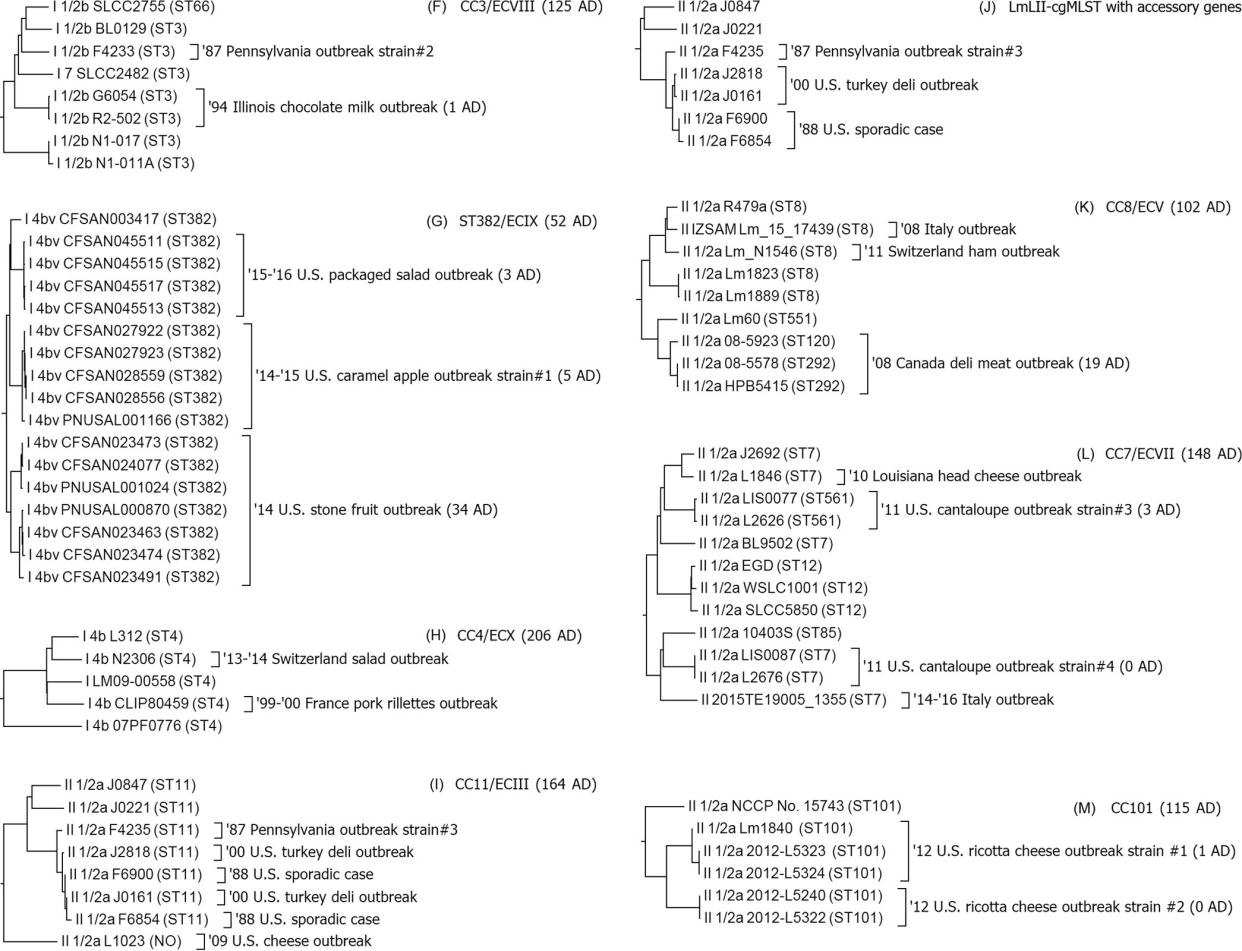
(A to C, E to I, and K) Subtrees of CC1/ECI (A), CC6/ECII (B), CC2/ECIV (C), CC5/ECVI (E), CC3/ECVIII (F), ST382/ECIX (G), CC4/ECX (H), CC11/ECIII (I), CC8/ECV (K), CC7/ECVII (L), and CC101 (M) by lineage-specific cgMLST schemes without accessory genes. (D) Subtree of the 1987-1989 UK pâté outbreak and outgroup determined by 1,701-cgMLST. The subtree has the same clustering as that obtained by 1,827-cgMLST. (J) Subtree of the 1988 U.S. sporadic case linked to hot dog, 2000 U.S. turkey deli meat outbreak, and outgroups by LmLII-cgMLST with the inclusion of accessory genes. Isolate identifiers are preceded by lineage and available serotype information and followed by the MLST ST in parentheses. The outbreak names are listed on the right side of the brackets. The maximum number of pairwise allele differences (ADs) among isolates of each outbreak strain, CC, or EC is listed in parentheses after their names in the subtrees of lineage-specific cgMLST without accessory genes. STs of three isolates (CFSAN025771 and CFSAN025772 in panel C and L1023 in panel I) were not observed (NO) in the Pasteur MLST database, but they differed from existing STs by one MLST allele and thus were assigned to CCs.

The L. monocytogenes lineage I-specific cgMLST (LmLI-cgMLST) had 2,400 core genes and 440 accessory genes (see Tables S2 and S3 in the supplemental material), and all genomes used to determine gene-set representativeness had ≥99% core genes. The L. monocytogenes lineage II-specific cgMLST (LmLII-cgMLST) had 2,342 core genes and 517 accessory genes (see Tables S4 and S5), and all genomes had ≥98.9% core genes. The L. monocytogenes lineage III-specific cgMLST (LmLIII-cgMLST) had 2,420 core genes and 491 accessory genes (see Tables S6 and S7), and all genomes had ≥96.2% core genes. We used the original definition of lineage III (2) and used the genome of a lineage IIIA strain (HCC23) as the reference for LmLIII-cgMLST. Thus, our LmLIII-cgMLST may not be perfectly representative for some of the lineage IIIB isolates. The analyses of major CCs or ECs are discussed below, with the number of MLST sequence types (STs), the number of MVLST virulence types (VTs), and the maximum number of pairwise allelic differences (ADs) being listed in the parentheses following each CC or EC. The maximum number of pairwise ADs among isolates of each outbreak strain is noted in the cgMLST trees (Fig. 2; see also Fig. S1 and S2 in the supplemental material). We measured the diversity of each clonal group by the maximum number of pairwise cgMLST allelic differences because no clear central allelic profiles of minimal spanning trees could be identified among isolates of some clonal groups or even among isolates of some outbreak strains (data not shown).

### (i) Lineage I CCs that have been associated with more than one outbreak and thus are also ECs (CCs/ECs).

#### (a) CC1/ECI (4 STs, 1 VT, 138 lineage-specific cgMLST ADs).

LmLI-cgMLST clustered previously identified CC1/ECI isolates (Table 1; Fig. 2A) from six outbreaks (a 1985 California cheese outbreak [21], a 1983-1987 Switzerland cheese outbreak [22], a 1976 France Anjou outbreak [23], a 1987 Pennsylvania outbreak [24], a 1981 Canada coleslaw outbreak [25], and a 2008 Austria jellied pork gastroenteritis outbreak [26]) and further identified the following to be part of CC1/ECI: isolates from a 2014-2015 U.S. caramel apple outbreak (27), an additional isolate (LL195) from the 1983-1987 Switzerland cheese outbreak, as well as isolates not known to be outbreak associated. NEdc2014, collected from food in the United States in 2014, had an allelic profile identical to that of the LL195 strain associated with the 1983-1987 Switzerland cheese outbreak. Other than this pair of identical isolates, our LmLI-cgMLST was able to differentiate individual outbreak strains and epidemiologically unrelated strains. SLCC2378 and WSLC1018 are lab derivatives of ATCC 19118 and formed a clade. Three clinical isolates from France (LM05-00008, LM05-00172, and LM07-00596) differed by up to 13 alleles, similar to the number of ADs among isolates of the same outbreak strain, but no information was available to determine whether these were epidemiologically related.

#### (b) CC6/ECII (1 ST, 1 VT, 167 ADs).

LmLI-cgMLST clustered previously identified CC6/ECII isolates (Table 1; Fig. 2B) from two outbreaks (a 1998 U.S. hot dog outbreak [17, 28] and a 2002 U.S. turkey deli meat outbreak [29, 30]) and further identified the following to be part of CC6/ECII: isolates from a 2013 U.S. multistate French-style cheese outbreak (31) and a clinical isolate from Italy (IZSAM_Lm_hs2008). LmLI-cgMLST placed isolates from the three outbreaks into 3 distinct clades and separated them from the unrelated IZSAM_Lm_hs2008.

#### (c) CC2/ECIV (5 STs, 2 VTs, 126 ADs).

LmLI-cgMLST clustered previously identified CC2/ECIV isolates (Table 1; Fig. 2C and D) from four outbreaks (a 1979 Boston, MA, vegetable outbreak [32], a 1983 Boston milk outbreak [33], a 1997 Italy corn gastroenteritis outbreak [34], and a 1987-1989 UK pâté outbreak [35]) and further identified the following to be part of CC2/ECIV: isolates from three outbreaks (a 1993-1994 Texas frozen vegetable outbreak [36], a 2014 U.S. multistate Hispanic-style cheese outbreak [37], and a 1983 Boston cheese outbreak [38]), as well as isolates not known to be outbreak associated. LmLI-cgMLST also placed isolates from different outbreaks into distinct clades and separated them from unrelated isolates. Isolates of the 1983 Boston milk and cheese outbreak had an MVLST VT that differed from the VT shared by other isolates by one allele, and thus, the 1983 Boston milk outbreak strain had been previously excluded from CC2/ECIV (4). However, LmLI-cgMLST, MLST (10), and kSNP-WGS (7) analyses all suggested that the 1983 Boston milk outbreak strain was part of CC2/ECIV. Isolates from each of the 1983 Boston milk and 1987-1989 UK pâté outbreak had multiple MLST STs but only one MVLST VT. Interestingly, LmLI-cgMLST placed a 1983 Boston cheese outbreak isolate (LI0521) (38) into the 1983 Boston milk outbreak clade. As these two outbreaks occurred in the same city during the same year and both were linked to dairy products, it is plausible that these outbreaks may have had a common source.

The 1987-1989 UK pâté outbreak was a common-source outbreak that lasted for more than 2 years (35), and isolates differed by up to 38 alleles, which suggested that they were more diverse than isolates from most of the other outbreaks. LmLI-cgMLST correctly clustered isolates from this outbreak into one clade in the neighbor-joining tree. In contrast, 1,701-cgMLST and 1,827-cgMLST placed Lm_1824, isolated from a cheese facility in Italy in 2012, inside the pâté outbreak clade (Fig. 2D; see also Fig. S1 and S2 in the supplemental material). Thus, the increased number of genes in LmLI-cgMLST offered improved epidemiological concordance over species-specific cgMLST.

#### (d) CC5/ECVI (1 ST, 1 VT, 242 ADs).

LmLI-cgMLST clustered previously identified CC5/ECVI isolates (Table 1; Fig. 2E) from a 2011 U.S. multistate cantaloupe outbreak (6) and further identified the following to be part of CC5/ECVI: isolates from three incidents (a 2013 U.S. Hispanic-style cheese outbreak [39], a 2010-2015 U.S. multistate ice cream outbreak [40], and a 2014 U.S. stone fruit [peach, nectarine, plum, etc.] recall [41]) as well as serotype 1/2b and 3b isolates not known to be outbreak associated. The diversity observed among isolates in this clonal group was slightly broader than that observed among isolates in other CCs/ECs. Isolates from two sources of the 2010-2015 U.S. multistate ice cream outbreak (40) differed by 186 to 195 alleles, indicating that these were, in fact, two different outbreak strains. A serotype 1/2b strain and a serotype IVb-v1 (4bv) strain were isolated from the stone fruit recall. Among them, the serotype IVb-v1 strain was linked to an outbreak as discussed below, but the serotype 1/2b strain was not linked to any reported illnesses (41). LmLI-cgMLST distinguished among isolates from individual outbreaks/incidents and epidemiologically unrelated isolates.

#### (e) CC3/ECVIII (2 STs, 1 VT, 125 ADs).

LmLI-cgMLST clustered previously identified CC3/ECVIII isolates (Table 1; Fig. 2F) from two outbreaks (a 1994 Illinois chocolate milk gastroenteritis outbreak [42] and a 1987 Pennsylvania outbreak [24]) and further identified the following to be part of CC3/ECVIII: an additional isolate (R2-502) from the chocolate milk gastroenteritis outbreak and isolates not known to be outbreak associated. LmLI-cgMLST also differentiated among the epidemiologically unrelated isolates.

#### (f) ST382/ECIX (1 ST, 1 VT, 52 ADs).

LmLI-cgMLST clustered ST382 isolates (Table 1; Fig. 2G) from three outbreaks (a 2014-2015 U.S. multistate caramel apple outbreak [27], a 2014 U.S. multistate stone fruit outbreak [41, 43], and a 2015-2016 U.S. multistate packaged leafy green salad outbreak [44]) as well as an isolate from monkey (CFSAN003417) in the United States. Therefore, we propose that these isolates be defined as a new EC, tentatively designated ECIX. It is a singleton and not a CC because no isolates that differed from ST382 by one MLST allele had been observed in the Pasteur MLST L. monocytogenes database as of August 2016 (9). Unlike outbreak strains from other CCs/ECs, isolates from the three outbreaks had the same pulsed-field gel electrophoresis (PFGE) profile (Phillip Curry, personal communication). LmLI-cgMLST also placed isolates from the three outbreaks into three distinct clades and separated them from CFSAN003417. All these isolates were serotype 4b by standard serotyping using antiserum agglutination but were serotype IVb-v1 (4bv) according to PCR-based serotyping (36, 45). ST382/ECIX was not observed in a large-scale MLST analysis of ∼2,000 L. monocytogenes isolates from multiple food, environmental, and clinical sources in several continents (1), nor was it observed in over 6,000 food and clinical L. monocytogenes isolates in France (14). Thus, ST382/ECIX appears to be an emerging clonal group strongly associated with produce commodities in the United States.

#### (g) CC4/ECX (1 ST, 1 VT, 206 ADs).

LmLI-cgMLST clustered isolates from two outbreaks (a 1999-2000 France pork rillettes outbreak [46] and a 2013-2014 Switzerland salad outbreak [47]) as well as isolates not known to be outbreak associated (Fig. 2H). Thus, we tentatively designated it as a new EC, ECX. CC4/ECX was one of the most dominant clones among clinical isolates in France and was shown to be most strongly associated with central nervous system or maternal-neonatal listeriosis in France (14). However, it has not been reported in any outbreaks outside Europe to date.

### (ii) Lineage II CC/EC.

#### (a) CC11/ECIII (2 STs, 1 VT, 164 ADs).

LmLII-cgMLST clustered previously identified CC11/ECIII isolates (Table 1; Fig. 2I and J) from four outbreaks/incidents (a 1987 Pennsylvania outbreak [24], a 2009 U.S. multistate Mexican-style cheese outbreak [48], a 1988 U.S. sporadic case [19], and a 2000 U.S. multistate turkey deli meat outbreak [49]) and CC11/ECIII isolates not known to be outbreak associated. Both the 1988 U.S. sporadic case and the 2000 U.S. multistate outbreak were linked to foods produced in the same facility in the southern United States (49). Although LmLII-cgMLST placed isolates from the 1988 U.S. sporadic case and the 2000 U.S. turkey deli meat outbreak into a clade, isolates from the two incidents did not form distinct subclades (Fig. 2I), similar to the clustering generated by 1,827-cgMLST or 1,701-cgMLST (see Fig. S1 and S2 in the supplemental material). The isolates from these two incidents had persisted in the same facility for over 11 years, and only 1 to 8 single nucleotide polymorphisms accumulated in the backbone of the genome, but significant divergence existed in their prophage regions (50). We thus explored LmLII-cgMLST with the inclusion of accessory genes which allowed us to differentiate between the two incidents (Fig. 2J), and many allelic differences were observed in the prophage regions (data not shown). This confirms suggestions from Orsi et al. (50), Verghese et al. (51), and Chen and Knabel (52) that prophage regions could be excellent markers for studying the epidemiology of L. monocytogenes. Because prophage regions are very diverse even among isolates from the same clonal group, the species-specific cgMLST gene set did not include the majority of those prophage regions (see Table S1 in the supplemental material) (12), which explains their failure to differentiate these two incidents. However, due to the recombinogenic nature of prophage regions, we caution that the inclusion of accessory genes should be explored only when analyzing a group of genetically close isolates that could not be distinguished on the basis of core genome diversity because the inclusion of recombinogenic regions could potentially confound the phylogenetic analysis of distantly related isolates.

CC11/ECIII was one of the least prevalent CCs among food and clinical isolates in France and was not analyzed in detail to explore its association with maternal-neonatal listeriosis (14). However, CC11/ECIII had been associated with three U.S. outbreaks: the 2000 multistate outbreak linked to deli meat produced in the southern United States primarily involving pregnant, newborn, and elderly patients (49), the 2009 multistate outbreak linked to Mexican-style cheese produced in the midwestern United States primarily involving pregnant women (48), and the 1987 Pennsylvania outbreak primarily involving newborn, elderly, and immunocompromised patients (24). This suggests that there is a persistent distribution of CC11/ECIII in the United States, and its association with maternal-neonatal listeriosis seems clear.

#### (b) CC8/ECV (4 STs, 1 VT, 102 ADs).

LmLII-cgMLST clustered previously identified CC8/ECV isolates (Table 1; Fig. 2K) from a 2008 Canada deli meat outbreak (53) and those not known to be outbreak associated and further identified the following to be part of CC8/ECV: isolates from two outbreaks (a 2011 Switzerland ham outbreak [54] and a 2008 Italy outbreak) as well as isolates not known to be outbreak associated. LmLII-cgMLST placed HPB5415 (55), previously not identified to be outbreak associated, in the 2008 Canada deli meat outbreak clade; it differed from a confirmed outbreak isolate (08-5578) by one LmLII-cgMLST allele. HPB5415 was isolated from meat during the investigation of the 2008 deli meat outbreak in Canada and had the PFGE profile of the outbreak isolate (55), which further supported the LmLII-cgMLST finding that HPB5415 was part of the 2008 Canada deli meat outbreak. Two isolates (Lm_1823 and Lm_1889) recovered from a cheese processing facility in Italy (56) differed by only one allele and therefore were the same strain.

#### (c) CC7/ECVII (4 STs, 3 VTs, 148 ADs).

LmLII-cgMLST clustered previously identified CC7/ECVII isolates (Table 1; Fig. 2L) from two outbreaks (a 2011 U.S. multistate cantaloupe outbreak [6] and a 2010 Louisiana head cheese outbreak [57]) and CC7/ECVII isolates not known to be outbreak associated and further identified the following to be part of CC7/ECVII: an isolate from a 2014-2016 Italy outbreak as well as isolates not known to be outbreak associated. Isolates from the 2011 U.S. cantaloupe outbreak formed two clades that differed from each other by 127 to 128 alleles, and thus, they were two different strains. Another CC7/ECVII clade included isolates SLCC5850, EGD, and WSLC1001 that differed by up to 17 alleles. Among them, SLCC5850 and EGD were lab derivatives of isolates associated with an outbreak among rabbits in the UK in 1924 (15, 58, 59), and WSLC1001, isolated in the UK in 1935, had previously been found to be highly similar to EGD (60), but no information on its relatedness to SLCC5850 or EGD was available. This clonal group contained 3 MVLST VTs, a finding which does not agree with the use of identical VTs to define ECs (4). CC7/ECVII was not among the top 12 most prevalent clones (14) of food and clinical isolates in France; however, this clonal group was associated with two U.S. multistate outbreaks and one outbreak in Italy, which further suggests the geographical difference in the distribution of CCs or ECs of L. monocytogenes.

### (iii) Other CCs associated with only one outbreak or not associated with any outbreaks.

In addition to the identification of CCs/ECs distributed worldwide, lineage-specific cgMLST schemes successfully clustered isolates from other MLST-defined CCs or singletons (Table 1) in a way similar to 1,827-cgMLST or 1,701-cgMLST (Fig. 1; see also Fig. S1 and S2 in the supplemental material). For example, CC101 included two outbreak strains from a 2012 U.S. multistate ricotta salata cheese outbreak which differed by 75 to 76 LmLII-cgMLST alleles (Fig. 2M). Among them, the clinical isolate and food isolate from the United States and a food isolate (Lm_1840) collected from the implicated facility in Italy (56) belonged to one outbreak strain (Fig. 2M). The diversities of most of the other CCs were similar to those described above, except that four CCs were notably more diverse than the others. The two CC19 isolates differed by 1 MLST allele and 297 LmLII-cgMLST alleles. The three CC31 isolates had an identical MLST ST but differed by up to 423 LmLII-cgMLST alleles. Among the three CC193 isolates, LM07456 differed from LM07425 and LM07424 by 1 MLST allele and 455 to 468 LmLII-cgMLST alleles. Among the three CC29 isolates, SLCC3287 differed from L2625 and BL0108 by 1 MLST allele and 673 to 680 LmLII-cgMLST alleles. Thus, isolates in MLST-defined CCs could have very large genetic diversity at the genome level. Nonetheless, the cgMLST clusters were still congruent with the CC identification; no isolates from other CCs were placed in the clade of each of these four CCs.

It is notable that while cgMLST correctly clustered isolates from each MLST-defined clonal group, the cgMLST subclades within each clonal group did not necessarily correspond to MLST STs because isolates of different STs could be in the same subclades and isolates of the same ST could be in different subclades (Fig. 2A, C, and L). Isolates of the same outbreak strain could have more than one ST (Fig. 2C and K). In addition, the number of MLST STs in each group of isolates was not an indication of the genome-level diversity. For example, CC2/ECIV isolates in this study (up to 126 LmLI-cgMLST ADs) had five MLST STs (Fig. 2C), but CC5/ECVI isolates (up to 242 LmLI-cgMLST ADs) had only one ST (Fig. 2E). Similarly, CC403 isolates (up to 9 LmLII-cgMLST ADs) had two MLST STs, but CC31 isolates (up to 423 LmLII-cgMLST ADs) had only one ST (Table 1).

### Isolates from common-source listeriosis outbreaks had various degrees of diversity.

Available information showed that in this study all outbreak strains represented by more than one isolate were from common-source outbreaks. The 2010-2015 U.S. multistate ice cream outbreak involved two facilities, but these were linked to two different clusters of illnesses. Isolates from the same outbreak could be different strains, some of which belonged to different clonal groups, e.g., the 2014-2015 caramel apple outbreak (CC1/ECI and ST382/ECIX), the 2009-2010 Austria/Germany cheese outbreak (CC398 and CC403), the 2011 U.S. cantaloupe outbreak (CC5/ECVI, CC7/ECVII, and CC29), and the 2005 Switzerland cheese outbreak (CC18 and CC26). Isolates from the same outbreak could also be different strains of the same clonal group, e.g., those from the 2012 U.S. ricotta salata cheese outbreak (CC101). These different strains from the same outbreak could have entered the related processing facilities through separate contamination events. In contrast, the maximum number of pairwise ADs among isolates associated with the 2002 U.S. multistate turkey deli meat outbreak, the 1987-1989 UK pâté outbreak, the 2014 stone fruit outbreak, and the 2010-2015 ice cream outbreak (strain number 2) were much lower (46, 38, 34, and 22 LmLI-cgMLST ADs, respectively [Fig. 2] and 30, 31, 24, and 16 1,701-cgMLST ADs, respectively [see Fig. S2 in the supplemental material]), suggesting that the processing facilities might have had a single contamination event, after which the contaminating strain accumulated genetic variations. This does not preclude the possibility that genetically close isolates entered the facility in separate events. In this study, we did not define isolates that were from the same outbreak but that differed by less than 50 lineage-specific cgMLST alleles as different strains. Our data could contribute to future updates of the definition of a strain that may incorporate whole-genome sequence similarities.

The diversity of isolates from these outbreaks appeared to be similar to that of some of the epidemiologically unrelated isolates: Lm_N1546 (2011 Switzerland ham outbreak) and IZSAM_Lm_15_17439 (2008 Italy outbreak), differing by 34 LmLII-cgMLST alleles (Fig. 2K) and 26 1,701-cgMLST alleles (see Fig. S2 in the supplemental material); Lm60 (2006 Switzerland clinical case) and 08-5578 (2008 Canada deli meat outbreak), differing by 49 LmLII-cgMLST alleles (Fig. 2K) and 34 1,701-cgMLST alleles (see Fig. S2); and CFSAN003417 (from a monkey) and PNUSAL000870 (2014 U.S. stone fruit outbreak), differing by 27 LmLI-cgMLST alleles (Fig. 2G) and 21 1,701-cgMLST alleles (see Fig. S2). For other outbreaks analyzed in this study, isolates of the same outbreak strain differed by up to 19 lineage-specific cgMLST alleles and 12 1,701-cgMLST alleles. In contrast, the epidemiologically unrelated isolates F4235 (1987 Pennsylvania outbreak) and J0161 (2000 U.S. multistate turkey deli meat outbreak) differed by up to 13 LmLII-cgMLST alleles (Fig. 2I) and 9 1,701-cgMLST alleles (see Fig. S2), but F4235 was placed outside the 2000 U.S. outbreak clade in the neighbor-joining tree by either cgMLST scheme. Therefore, when attempting to define a listeriosis outbreak, the number of allelic differences should always be combined with cgMLST clustering and with epidemiological evidence.

In this study, the sequences obtained from NCBI or ENA were probably sequenced and assembled by different platforms and software. In future, in order to precisely determine the genomic diversity among isolates of a clonal group or outbreak strain, it is preferable that isolates be sequenced and assembled using the same protocol. Nonetheless, our analyses using the same cgMLST scheme(s) provided a measure of relative diversity among isolates of each clonal group and isolates of each outbreak strain. Isolates of the same outbreak strain were sequenced by different research groups but were still clustered by cgMLST, indicating that cgMLST is tolerant of the interlaboratory variability of WGS.

In this study, we used *in silico* MLST to assign clonal groups but also performed *in silico* MVLST, which allowed partial comparison between MLST and MVLST. Isolates of the same CC/EC had an identical MVLST VT, except that multiple VTs differing by one allele were observed among isolates in CC2/ECIV and CC7/ECVII. We noted that it was possible for isolates of the same CC/EC or outbreak strain to have one VT but more than one MLST ST. This is consistent with the findings of a previous study showing that the 7-gene MLST had a discriminatory power similar to that of the 8-gene MvLST, which combined the target regions of the 6-gene MVLST with two additional full-length virulence genes (10) and thus possessed an increased discriminatory power over that of the 6-gene MVLST.

### Conclusions.

cgMLST could identify clonal groups and differentiate outbreak strains from epidemiologically unrelated strains of L. monocytogenes. Lineage-specific cgMLST increased the discriminatory power over that of the species-specific cgMLST. Isolates of different clonal groups or outbreak strains had various degrees of genome-level diversity.

## Supplementary Material

Supplemental material
