# Supplementary material for: Core Genome Multilocus Sequence Typing for Identification of Globally Distributed Clonal Groups and Differentiation of Outbreak Strains of Listeria monocytogenes
Source: Appl Environ Microbiol. 2016 Sep 30;82(20):6258–72. doi: 10.1128/AEM.01532-16 (PMC5068157; doi:10.1128/AEM.01532-16)
Supplement: Supplemental material [file supp_82_20_6258__index.html]

Supplemental material 

# Core Genome Multilocus Sequence Typing for Identification of Globally Distributed Clonal Groups and Differentiation of Outbreak Strains of Listeria monocytogenes

## Supplemental material

- Supplemental file 1 -

  Neighbor-joining trees of all the isolates using 1827-cgMLST (Fig. S1) and 1701-cgMLST (Fig. S2).

  PDF, 8.1M
- Supplemental file 2 -

  Core gene set of Lm-cgMLST (1827-cgMLST) (Table S1).

  XLS, 308K
- Supplemental file 3 -

  Core gene set of LmLI-cgMLST (Table S2).

  XLS, 343K
- Supplemental file 4 -

  Accessory gene set of LmLI-cgMLST (Table S3).

  XLS, 76K
- Supplemental file 5 -

  Core gene set of LmLII-cgMLST (Table S4).

  XLS, 375K
- Supplemental file 6 -

  Accessory gene set of LmLII-cgMLST (Table S5).

  XLS, 90K
- Supplemental file 7 -

  Core gene set of LmLIII-cgMLST (Table S6).

  XLS, 345K
- Supplemental file 8 -

  Accessory gene set of LmLIII-cgMLST (Table S7).

  XLS, 82K
