# Supplementary material for: Core Genome Multilocus Sequence Typing for Identification of Globally Distributed Clonal Groups and Differentiation of Outbreak Strains of Listeria monocytogenes
Source: Appl Environ Microbiol. 2016 Sep 30;82(20):6258–72. doi: 10.1128/AEM.01532-16 (PMC5068157; doi:10.1128/AEM.01532-16)
Supplement: Supplemental material [file AEM.01532-16_zam999117471so1.pdf]

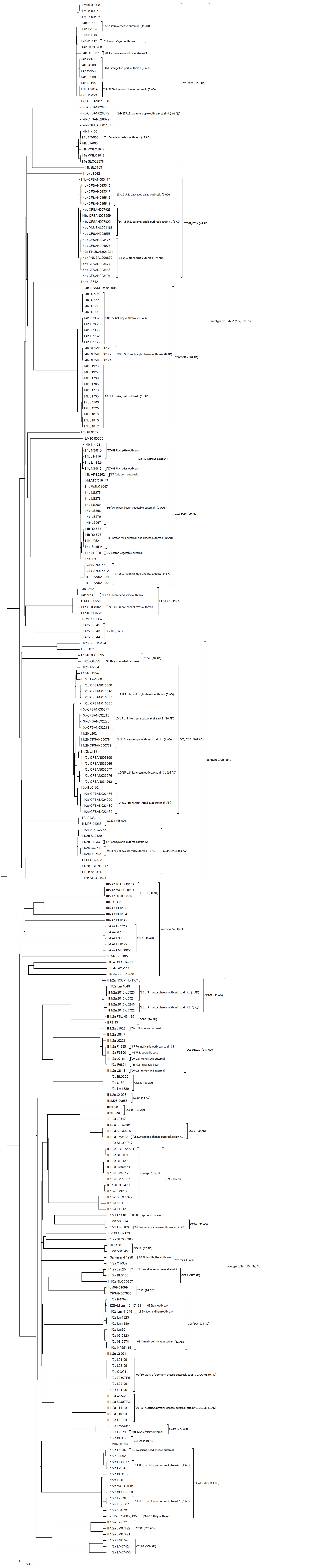

Figure S1. 1827-cgMLST analysis of all 258 isolates. The outbreak, clonal complex, epidemic clone and serotype information are on the right side of the brackets. The maximum number of pairwise allelic differences (AD) of each clonal group or isolates of the same outbreak strain is listed in the parenthesis.

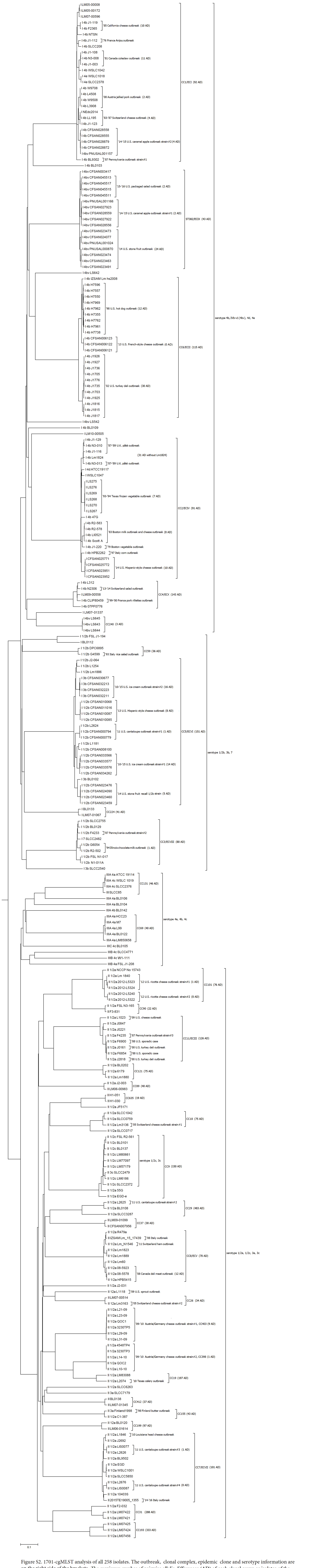

Figure S2. 1701-cgMLST analysis of all 258 isolates. The outbreak, clonal complex, epidemic clone and serotype information are on the right side of the brackets. The maximum number of pairwise allelic differences (AD) of each clonal group or isolates of the same outbreak strain is listed in the parenthesis.
